# Salivary Aldehyde Dehydrogenase: Activity towards Aromatic Aldehydes and Comparison with Recombinant ALDH3A1

**DOI:** 10.3390/molecules14072363

**Published:** 2009-07-02

**Authors:** Joanna Giebułtowicz, Renata Wolinowska, Anna Sztybor, Monika Pietrzak, Piotr Wroczyński, Jacek Wierzchowski

**Affiliations:** 1 Department of Drugs Analysis, Faculty of Pharmacy, Medical University of Warsaw, 1 Banacha Street, PL-02-097, Warsaw, Poland; E-mail: joanna.giebultowicz@gmail.com (J.G.); 2 Department of Pharmaceutical Microbiology, Faculty of Pharmacy, Medical University of Warsaw, 3 Oczki Street, PL-02-007, Warsaw, Poland; E-mail: rwolinowska@wum.edu.pl (R.W.); 3 Department of Biophysics, University of Warmia and Mazury, 4 Oczapowskiego St., PL-10-719 Olsztyn, Poland

**Keywords:** saliva, aldehyde dehydrogenase, enzyme kinetics, aromatic aldehydes

## Abstract

A series of aromatic aldehydes was examined as substrates for salivary aldehyde dehydrogenase (sALDH) and the recombinant ALDH3A1. Para-substituted benzaldehydes, cinnamic aldehyde and 2-naphthaldehydes were found to be excellent substrates, and kinetic parameters for both salivary and recombinant ALDH were nearly identical. It was demonstrated that for the fluorogenic naphthaldehydes the only produced reaction product after incubation in saliva is the carboxylate.

## 1. Introduction

**Scheme 1:**
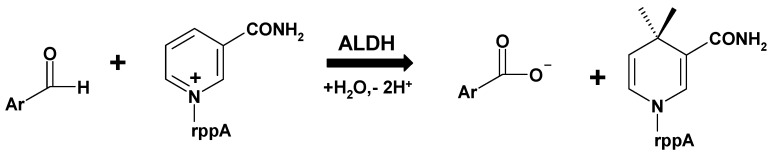
Enzymatic oxidation of aryl aldehydes, catalyzed by ALDH.

Human saliva exhibits significant activity of many detoxifying enzymes, including aldehyde dehydrogenase (ALDH, E.C.1.2.1.3, cf. Scheme 1), which presumably protects the organism from various aldehydes contained in food either as natural ingredients or as contaminants [1,2,3,4]. Salivary aldehyde dehydrogenase probably consists of one isozyme, classified as ALDH3A1 [4,5], virtually inactive toward acetaldehyde, but active toward aromatic and long aliphatic aldehydes, including the most toxic 4-hydroxy-2-nonenal [5,6,7,8], formed during the process of lipid autooxidation. This dimeric isozyme is selectively expressed in various human organs, the highest activities found in eye cornea, stomach and lungs [9]. Occasionally, it undergoes overexpression in neoplastic tissues [5,10], leading to increased resistance to oxazaphosphorine chemotherapy [11]. Still another ALDH isozyme, known as ALDH6 or ALDH1A3, which is tetrameric, has been found in the salivary glands [12,13], but its participation in the salivary ALDH activity remains uncertain.

Substrate specificity of the recombinant human ALDH3A1 has been previously studied by Pappa *et al*. [14], who indicated benzaldehyde as the best substrate (in terms of V_max_), and saturated aliphatic aldehydes as exhibiting the highest V_max_/K_m_ values, due to very low K_m_. However, no aromatic aldehydes, except for benzaldehyde, were examined.

We have previously shown that some highly fluorogenic aromatic aldehydes, particularly substituted 2-naphthaldehydes, are excellent substrates for salivary ALDH [15,16], allowing sensitive fluorimetric detection of its activity, applicable for population studies [17,18]. These investigations are important due to known variability of the salivary ALDH activity [18], and may be useful for food safety and nutrition research. We presently compare salivary ALDH activity towards a series of aromatic aldehydes (Scheme 1), including those known as food components, with that of the recombinant ALDH3A1 enzyme, with the aim of identifying isozyme(s) responsible for the salivary activity and to further characterize their substrate preferences.

An additional goal of this work was to check the validity and specificity of the naphthaldehyde-based fluorimetric assay of the ALDH activity [15], in particular with a view to application in human saliva studies. We here address two questions, related to saliva reaction with the naphthaldehydes: (a) are there any other fluorescent products of the reaction except the carboxylates; and (b), do the reaction kinetics agree with that of the purified ALDH3A1.

## 2. Results and Discussion

### 2.1. Salivary ALDH activity – apparent kinetics and product analysis

As shown previously, human saliva is able to oxidize a variety of aromatic aldehydes, like benzaldehyde, and various naphthaldehyde derivatives [16], and this activity has been ascribed to a dimeric ALDH3A1 isozyme [1,5]. The aromatic substrates, particularly highly fluorogenic 2-naphthaldehydes, like 6-methoxy-2-naphthaldehyde (MONAL) and 6-dimethylamino-2-naphthaldehyde (DANAL), have been previously reported as excellent salivary ALDH substrates [15,16]. Their oxidation was characterized by submicromolar or low micromolar apparent K_m_ values [16], and high catalytic constants, comparable to that of benzaldehyde (see Table 1). In contrast, 1-naphthaldehydes were almost completely inactive [16], although they occasionally reacted chemically with saliva. Benzaldehyde and its derivatives are also good substrates for salivary ALDH, and their oxidation can be followed using the increase of the NADH fluorescence at 460 nm. Using this method, we have found that both anisaldehyde (4-methoxybenzaldehyde) and cinnamic aldehyde (trans-3-phenylacrylaldehyde) are excellent substrates for salivary ALDH (see Table 1), with the latter compound exhibiting a catalytic constant (k_cat_) almost twice as large as that for benzaldehyde.

**Figure 1:**
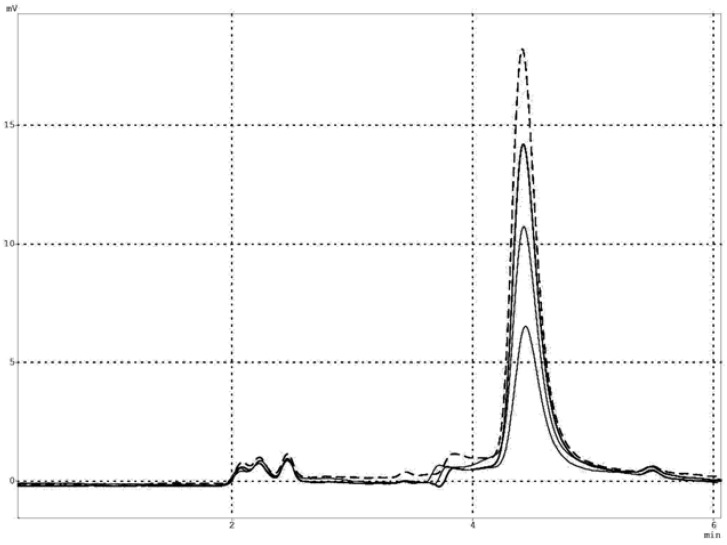
HPLC elution profiles for oxidation of aromatic aldehydes with salivary ALDH, recorded with fluorimetric detector at 360 nm, with excitation at 315 nm. Reaction time was 5 (lowest curve), 10, 15 and 20 minutes (----).

To cross-check the validity of the foregoing results, obtained using fluorimetric method with crude, diluted saliva [13,15], we analyzed products of MONAL transformation by saliva samples in the presence of 100 μM NAD^+^ using HPLC with fluorimetric detection. A typical HPLC profile of the reaction mixture after 5-20 min of reaction at 25°C, recorded using a fluorescence detector, is presented in Figure 1. It is evident that the carboxylate is the only fluorescent product of this reaction and its concentration linearly depends on reaction time, thus supporting our previous kinetic analysis. Further support comes from comparison of the kinetic parameters obtained for reactions catalyzed by salivary ALDH and purified recombinant ALDH3A1 (see next section). 

### 2.2. Kinetic properties of ALDH3A1 towards aromatic aldehydes – comparison with salivary ALDH

The kinetic oxidation parameters of several aldehydes by the recombinant ALDH3A1 have been determined using fluorimetric and/or spectrophotometric methods. The purified enzyme has been found active towards a series of *para*-substituted benzaldehydes and 2-naphthaldehydes, but was virtually inactive towards substituted 1-naphthaldehydes. Comparison of kinetic parameters estimated for the recombinant enzyme and salivary ALDH is shown in Table 1.

**Figure 2:**
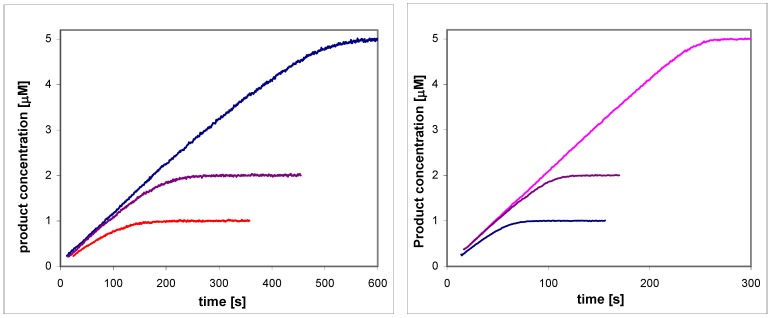
Reaction progress curves, recorded fluorimetrically, for enzymatic oxidation of 2-naphthaldehde (left) and MONAL (right) with the recombinant ALDH3A1 as catalyst. Initial naphthaldehyde concentrations were 5, 2 and 1 μM. Concentration of NAD^+^ was 100 μM.

**Figure 3:**
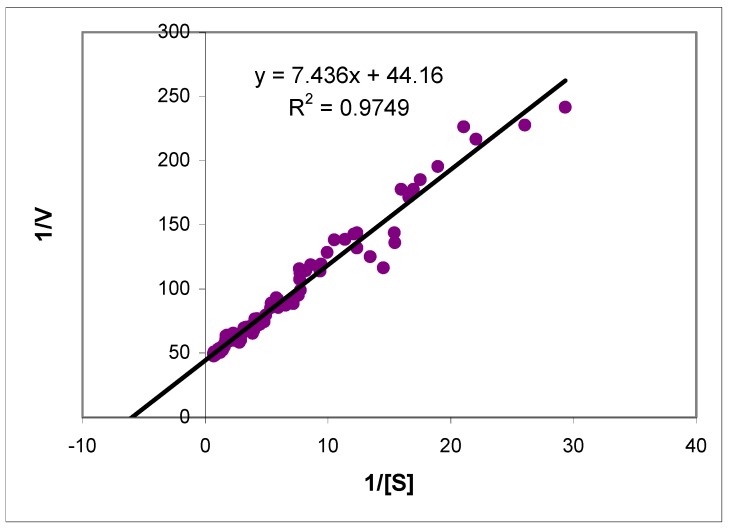
The Lineweaver-Burk plot for the enzymatic oxidation of 2 μM MONAL catalyzed by the recombinant ALDH3A1. Rates were obtained by numerical differentiation of the progress curve from Fig. 2. The fitted K_m_ is 0.16 μM.

The most interesting are the submicromolar K_m_ values for enzymatic oxidation of 2-naphthaldehyde and MONAL. Such low K_m_ values, previously reported for the same, and isomeric naphthaldehydes oxidized by ALDH1A1 from the erythrocytes and human liver [19], and recently for oxidation of pyrene aldehydes with ALDH3A1 [20], are hardly measurable using standard spectrophotometric procedures, but may be estimated from reaction progress curves with variable initial concentration of the substrate (cf. Figure 2 and Figure 3), thanks to highly fluorogenic behaviour of the naphthaldehydes [15].

It is evident that aromatic aldehydes, particularly those with extended aromatic systems and/or electron donating substituents, bind tightly to the enzyme and are effectively oxidized. The best substrates are cinnamic aldehyde, with a V_max_ twice as large as that for benzaldehyde, and 2-naphthaldehyde, exhibiting the highest V_max_/K_m_. There is considerable selectivity in substrate geometry, since various substituted 1-naphthaldehydes are not oxidized, and 1-naphthaldehyde is oxidized very slowly (Table 1), undoubtedly due to steric factors within the binding site. By contrast, long aliphatic unsaturated aldehydes, like *trans*-hexenal, *trans*-octenal, and acrolein, which are good substrates for ALDH3A1, are characterized by much higher K_m_ values, typically >100 μM [14].

Kinetic parameters of the reaction, particularly K_m_ values, obtained for ALDH3A1, are virtually identical to those previously measured for salivary ALDH [16,18], thus confirming that at least in relation to aromatic aldehydes, the salivary ALDH activity is exclusively due to the ALDH3A1 isozyme. This also constitutes final proof of the validity of the fluorimetric method of the salivary ALDH detection, based on oxidation of the fluorogenic naphthaldehydes [15,16,17,18].

**Table 1:** Kinetic parameters, determined spectrophotometrically or fluorimetrically, for enzymatic oxidation of aromatic aldehydes by recombinant ALDH3A1, compared to those for salivary ALDH. Maximal rates are measured relative to that of benzaldehyde (100).

Aldehyde	Recombinant ALDH3A1		salivary ALDH		
	K_m_ [μM]	V_max_ [relative]	K_m_ [μM]	V_max_ [relative]	_obs_ [nm] (Δ [M^-1^cm^-1^])
Benzaldehyde	148	100	160^a^	100	340 (6,200)
4-methoxybenzaldehyde (anisaldehyde)	19	73	nd	~45	340 (6,200)
4-dimethylaminobenzaldehyde	4.2	8	nd	nd	350 (-22,000)
4-hydroxy-3-methoxy-benzaldehyde (vanillin)	155	6	nd	nd	310 (-7,000)
cinnamic aldehyde	6	160	nd	190	340 (6,200)
2-naphthaldehyde	0.4	101	0.46^a^	105	330 (3,900)
6-methoxy-2-naphthaldehyde (MONAL)	0.16	47	0.2^a^	52	315 (-7,600)
6-dimethylamino-2-naphthaldehyde (DANAL)	~20	~21	7.2^a^	21^a^	380 (-11,000)
1-naphthaldehyde	nd	~1.5	-	<25^b^	320 (-4,000)
4-methoxy-1-naphthaldehyde	-	<1	-	<8^a^	360 (-9,000)
4-dimethylamino-1-naphthaldehyde	-	<1	-	<9^a^	400 (-10,000)
7-methoxy-1-naphthaldehyde	-	<1	-	<1^a^	360 (-4,000)

^a^ Data from refs [13,15], determined fluorimetrically, cross-checked in this work; ^b^ This substrate reacted chemically with saliva constituents giving unstable fluorescence background.

It is important to notice that some of the examined aldehydes are natural constituents of food, particularly of plant origin [2]. This refers, in particular, to anisaldehyde, benzaldehyde, and cinnamic aldehyde, all three being excellent substrates for ALDH3A1, and to vanillin, which is a moderate substrate. The postulated role of the salivary ALDH in protection against these highly reactive aldehydes, suspected as risk factors in the development of cancer of the digestive tract [6,7,8,10,20,21] is therefore plausible. Furthermore, the same isozyme, when expressed in neoplastic cells, is known to impair the oxazaphosphorine chemotherapy by inactivating the key intermediate aldophosphamide [4,11,22,23,24], and a mutated form of ALDH3A1 has been recently reported to increase risk of haemorrhagic cystitis during the chemotherapy [25]. Therefore, the detailed kinetic characterization of ALDH3A1 may be useful for further pharmacological studies.

## 3. Experimental

### 3.1. General

Benzaldehyde, 4-dimethylaminobenzaldehyde, 1- and 2-naphthaldehydes, 6-methoxy-2- naphthaldehyde, 4-methoxy-1-naphthaldehyde, 4-dimethylamino-1-naphthaldehyde and the corresponding carboxylic acids were from Aldrich. Anisaldehyde, cinnamic aldehyde and vanillin were purchased from Sigma. DANAL and 7-methoxy-1-naphthaldehyde were synthesized previously [19]. Coenzymes NAD^+^ and NADH, as well as dithiothreitol, were from Sigma. All other chemicals were of analytical grade.

All assays were run in 50 mM phosphate buffers, pH 7.5, at 25°C, in the presence of 0.5 mM EDTA and 0.5 mM DTT. Concentration of NAD^+^ was kept constant at 100 μM. Fluorimetric assays were run on a thermostated Perkin-Elmer LS-50B instrument. Instrumental settings for DANAL oxidation were: excitation wavelength, 320 nm, emission monitored at 420 nm, and for 2-naphthaldehyde and MONAL 315 nm and 360 nm, respectively, with spectral bandwidths 7 and 10 nm for the excitation and emission beams. For non-fluorescent carboxylates, fluorescence of NADH was followed at 460 nm, with excitation at 340-350 nm, and spectral resolution of 10-15 nm. Purified reaction products (carboxylates or NADH) at the concentrations of 2-5 μM were used as internal standards in the fluorimetric assays to obtain absolute reaction rates, which were calculated according to the formula [15]:

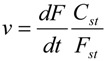

where C_st_ is standard concentration, F_st_ its fluorescence and dF/dt slope of the fluorescence time-dependence.

UV absorption was measured using a Cary 219 spectrophotometer. Optimum wavelengths and Δ values for oxidation of aromatic aldehydes with NAD^+^ were determined and collected in Table 1. Kinetic parameters for enzymatic reactions were calculated according to Lineweaver-Burke standard procedure, except those cases where K_m_ values were very low, where progress reaction curve analysis was applied.

HPLC was performed on a Shimadzu chromatograph consisting of a LC-10AD pump and RF10AXL fluorescence detector. Twenty microliters of the deproteinized incubation mixtures were introduced to the column. Separation was performed on the Supelcosil LC-18-DB 25 cm × 4.6 mm, 5 µm column (Supelco) under isocratic conditions. The mobile phase consisted of an acetonitrile-water mixture 65:35 (v/v) at pH of 2.8. The mobile phase was pumped at a flow-rate of 1 mL/min. Chromatography was performed at 30 °C. Fluorescence excitation and emission wavelengths were set at 310 and 360 nm, respectively. All eluents were of HPLC purity grade.

### 3.2. Saliva collection

Human saliva samples were obtained from healthy adult volunteers, all of them declaring non-smoking and non-drinking behaviour. Saliva samples were collected before first meal, after thoroughly washing mouths, directly to test tubes containing cooled 50 mM phosphate buffer, pH 7.5, with addition of 0.5 mM EDTA and 0.5 mM DTT. Final dilution of the saliva with buffer was ca. 1:1. The saliva samples were centrifuged at 3,500 rpm for 5-10 minutes and the supernatant was gently collected and stored in ice [17]. After 1 – 5 hours of incubation the ALDH activity of the supernatant was measured after diluting 1:20 with buffer in the presence of the appropriate aldehyde and NAD^+^.

### 3.3. Cloning of the cDNA for ALDH3A1 and its overexpression

The full- length human ALDH3A1 gene was PCR amplified from “TrueClone”, cDNA clone in pCMV6-AC vector purchased in OriGene (Rockville, MD, U.S.A.). The sequence of the 5’ and 3’ PCR primers were: 5’CTAGCTAGCATGAGCAAGATCAGCGAGGCC3’ and 5’CCGGAATTCTC AGTGCTGGGTCATCTTGGC 3’, respectively.

**Figure 4:**
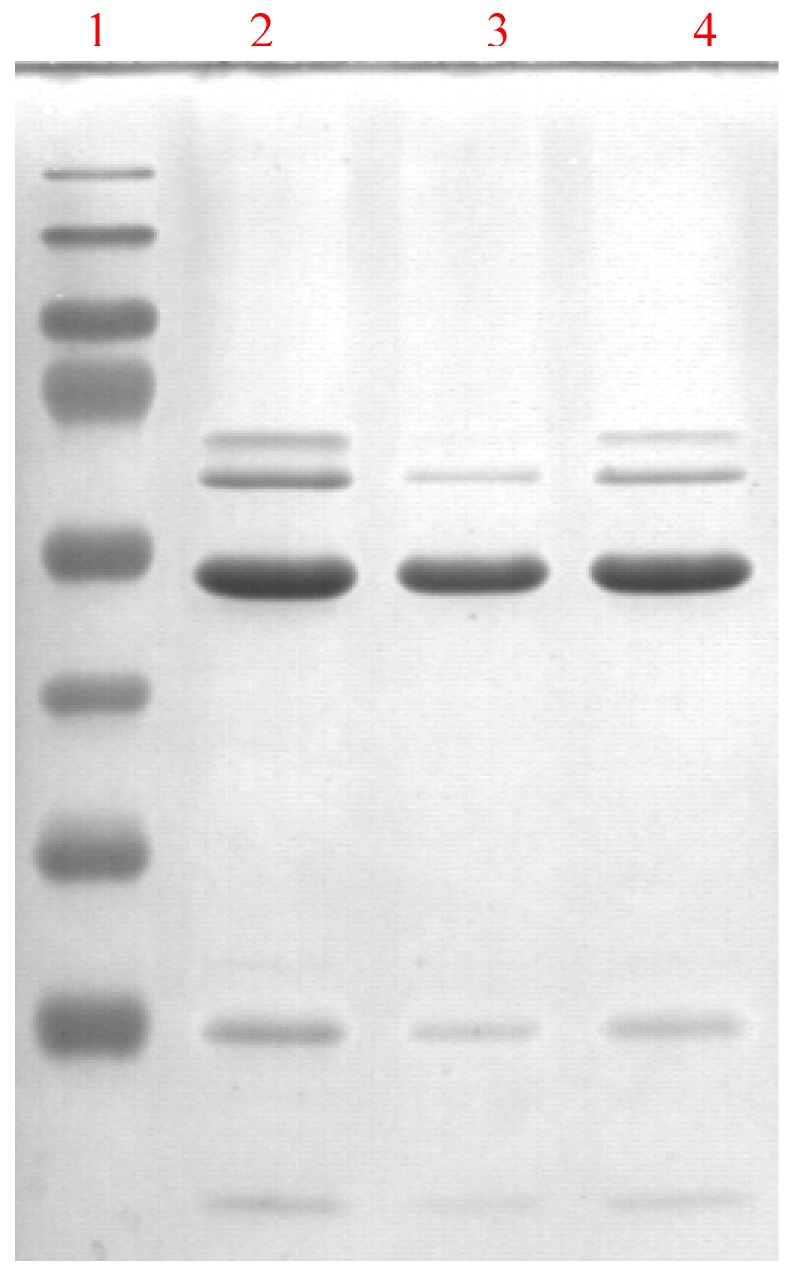
SDS/PAGE of recombinant ALDH3A1 after purification: lane 1, PageRuler (Fermantas) molecular mass marker proteins (from bottom to top: 26, 34, 43, 55, 72, 95, 130, 170 kDa); lane 2, solution from the first elution; lane 3, solution from the second elution; lane 4, eluate after dialysis. The gel was stained with Coomassie Brilliant Blue.

A *Nhe*I site was introduced by the PCR primer on the 5’ end, whereas a *EcoR*I site on 3’ end. The resulting 1.5-kb PCR-amplified fragment was digested with NheI and EcoRI, gel-purified using the QIAquick Gel Extraction Kit (Qiagen), and ligated with pET-28a (Novagene) that had been digested with the same enzymes and gel-purified. The ligation reaction was used to transform *E.coli*BL21(DE3) competent cells (Invitrogen). The sequence of the entire inset of the pET28a-ALDH3A1 plasmid was verified by sequencing. 

The cultures of the overproducing stain were grown at 37°C in LB broth (35 g/L tryptone, 20 g/L yeast extract, 5g/L NaCl) supplemented with 50 µg/mL kanamycin to an OD_600_ of 0.6. The expression was induced by adding IPTG to a final concentration of 1 mM. Recombinant ALDH3A1 was isolated (see Figure 4) with Ni-NTA Fast Start Kit (Qiagen) and dialyzed overnight to 50 mM pyrophosphate buffer containing 1 mM EDTA and 1 mM DTT.

## 4. Conclusions

Aromatic aldehydes, particularly *para*-substituted benzaldehydes and 2-naphthaldehydes, are excellent substrates for both salivary ALDH and the recombinant ALDH3A1. The obtained K_m_ values for various aldehydes are virtually identical with both activities, confirming that ALDH3A1, as an isozyme particularly reactive toward aromatic aldehydes, is primarily responsible for the salivary ALDH activity. Consequently, full specificity of the fluorimetric assay of the salivary ALDH3A1, based on oxidation of the fluorogenic 2-naphthaldehydes, is confirmed.

## References

[1] Dyck L.E. (1995). Polymorphism of a class 3 aldehyde dehydrogenase present in human saliva and in hair roots. Alcohol. Clin. Exp. Res..

[2] Feron V.J., Til H.P., de Vrijer F., Woutersen R.A., Cassee F.R., van Bladeren P.J. (1991). Aldehydes: Occurence, carcinogenic potential, mechanism of action and risk assessment. Mutat. Res..

[3] Vasiliou V., Pappa A., Estey T. (2004). Role of human aldehyde dehydrogenases in endobiotic and xenobiotic metabolism. Drug Metab. Rev..

[4] Sládek N.E. (2003). Human aldehyde dehydrogenases: Potential pathological, pharmacological, and toxicological impact. J. Biochem. Mol. Toxicol..

[5] Sreerama L., Hedge M.W., Sladek N.E. (1995). Identification of a class 3 aldehyde dehydrogenase in human saliva and increased levels of this enzyme, glutathione S-transferases, and DT-diaphorase in the saliva of subjects who continually ingest large quantities of coffee or broccoli. Clin. Cancer Res..

[6] Ellis E.M. (2007). Reactive carbonyls and oxidative stress: Potential for therapeutic intervention. Pharmacol. Therap..

[7] Townsend A.J., Leone-Kabler S., Haynes R.L., Wu Y., Szweda L., Bunting K.D. (2001). Selective protection by stably transfected human ALDH3A1 (but not human ALDH1A1) against toxicity of aliphatic aldehydes in V79 cells. Chem. Biol. Interact..

[8] Pappa A., Chen C., Koutalos Y., Townsend A.J., Vasiliou V. (2003). ALDH3A1 protects human corneal epithelial cells from ultraviolet- and 4-hydroxy-2-nonenal-induced oxidative damage. Free Radical Bio. Med..

[9] Estey T., Piatigorsky J., Lassen N., Vasiliou V. (2007). ALDH3A1: a corneal crystallin with diverse functions. Exp. Eye Res..

[10] Jelski W., Szmitkowski M. (2008). Alcohol dehydrogenase (ADH) and aldehyde dehydrogenase (ALDH) in the cancer diseases. Clin. Chim. Acta.

[11] Sladek N.E. (1999). Aldehyde dehydrogenase-mediated cellular relative insensitivity to the oxazaphosphorines. Curr. Pharm. Des..

[12] Hsu L.C., Chang W.C., Hiraoka L., Hsieh C.L. (1994). Molecular cloning, genomic organization, and chromosomal localization of an additional human aldehyde dehydrogenase gene, ALDH6. Genomics.

[13] Aldehyde dehydrogenase gene superfamily database. www.aldh.org.

[14] Pappa A., Estey T., Manzer R., Brown D., Vasiliou V. (2003). Human aldehyde dehydrogenase 3A1 (ALDH3A1): Biochemical characterization and immunohistochemical localization in the cornea. Biochem. J..

[15] Wierzchowski J., Wroczynski P., Laszuk K., Interewicz E. (1997). Fluorimetric detection of aldehyde dehydrogenase activity in human blood, saliva and organ biopsies, and kinetic differentiation between class I and class III isozymes. Anal. Biochem..

[16] Wroczyński P., Wierzchowski J. (2000). Aromatic aldehydes as fluorogenic indicators for human aldehyde dehydrogenases and oxidases. Analyst.

[17] Wroczyński P., Wierzchowski J., Rakowska A., Chimkowska M., Targoński J. (2004). Aldehyde dehydrogenase in human saliva – evaluation of its oxidation status. Acta Pol. Pharm..

[18] Wierzchowski J., Pietrzak M., Szeląg M., Wroczyński P. (2008). Salivary aldehyde dehydrogenase – reversible oxidation of the enzyme and its inhibition by caffeine, investigated using fluorimetric method. Arch. Oral Biol..

[19] Wierzchowski J., Interewicz E., Wroczynski P., Orlanska I. (1996). Continuous fluorimetric assay for human aldehyde dehydrogenase and its application to blood analysis. Anal. Chim. Acta.

[20] Glatt H., Rost K., Frank H., Seidel A., Kollock R. (2008). Detoxification of promutagenic aldehydes derived from methylpyrenes by human aldehyde dehydrogenases ALDH2 and ALDH3A1. Arch. Biochem. Biophys..

[21] Seitz H.K., Matsuzaki S., Yokoyama A., Homann N., Vaekaevainen S., Wang X.D. (2001). Alcohol and cancer. Alcohol. Clin. Exper. Res..

[22] Sládek N.E., Kollander R, Sreerama L., Kiang D.T. (2002). Cellular levels of aldehyde dehydrogenases (ALDH1A1 and ALDH3A1) as predictors of therapeutic responses to cyclophosphamide-based chemotherapy of breast cancer: A retrospective study. Cancer Chemother. Pharmacol..

[23] Moreb S.J., Muhoczy D., Ostmark B., Zucali J.R. (2007). RNAi-mediated knockdown of aldehyde dehydrogenase class-1A1 and class-3A1 is specific and reveals that each contributes equally to the resistance against 4-hydroperoxycyclophosphamide. Cancer Chemother. Pharmacol..

[24] Ho K.K., Mukhopadhyay A., Li Y.F., Mukhopadhyay S., Weiner H. (2008). A point mutation produced a class 3 aldehyde dehydrogenase with increased protective ability against the killing effect of cyclophosphamide. Biochem. Pharmacol..

[25] Ekhart C., Rodenhuis S., Smits P.H.M., Beijnen J.H., Huitema A.D.R. (2008). Relations between polymorphisms in drug-metabolising enzymes and toxicity of chemotherapy with cyclophosphamide, thiotepa and carboplatin. Pharmacogenet. Genomics.

